# Pressure-Volume-Temperature Relations in Liquid and Solid Tritium

**DOI:** 10.6028/jres.098.044

**Published:** 1993

**Authors:** ER Grilly

**Affiliations:** Los Alamos National Laboratory, Los Alamos, NM 87545

**Keywords:** deuterium, hydrogen, *PVT* relations, tritium

## Abstract

*PVT* relations in liquid and solid T_2_ near the melting curve were measured over 20.5 K–22.1 K and 0 MPa–7 MPa (0 bar–70 bar) with a cell that used diaphragms for pressure and volume variation and measurement. Because of ortho-para self conversion, the melting pressure *P_m_* and the liquid molar volume *V*_lm_ increased with time. The rates were consistent with a second order reaction similar to that for *c* the *J* = odd concentration:

$$dc/dt=-k_{1}c^{2}+k_{2}c(1-c),$$

where *k*_1_ = 6−9×l0^−2^h^−1^. By extrapolation, the ortho and para forms differed by *ΔP_m_*~6 bar and *ΔV_lm_*~0.5%. Measurements of the volume change on melting and the thermal expansion and compressibility for liquid T_2_ were consistent with those for H_2_ and D_2_. Impurities such as H_2_, HT, DT, and ^3^He were removed by a technique using an adsorption column of cold activated alumina. Corrections for ^3^He growth during an experiment were adequate except near the triple point.

## 1. Introduction

Basic interest in the hydrogens H_2_, D_2_, and T_2_ is notably enhanced by the existence of significant zero-point energy, large relative mass differences, and different ortho-para characteristics. In addition, D_2_ and T_2_ in the condensed phases are prime candidates as fuels for controlled nuclear fusion.

Although the discoveries of D_2_ in 1931 [1] and T_2_ in 1934 [2] were close together in time, the pressure-volume-temperature (*PVT*) measurements on T_2_ have lagged far behind those on D_2_. Essentially they were the 1951 measurements of vapor pressure [3] and liquid density [4] up to 3 bar^1^ and 29K and the 1956 melting curve determination up to 3100 bar and 60 K [5]. Contributing to the sluggishness of research efforts have been the high cost of T_2_ and the difficulties that arise from its radioactivity (2.8 Ci/cm^3^ STP gas). Health and environment concerns require great care in containing T_2_ and definite provisions for accidental release. The continual creation of ^3^He from nuclear decay automatically adds a significant impurity. Self-heating demands proper equipment design and/or data corrections. The exchange of tritium with hydrogen in equipment causes physical breakdown of plastics and contamination of the tritium with hydrogen. These problems have affected the accuracy and completeness of the data reported here.

## 2. Apparatus and Procedures

The apparatus and procedures were basically those used for similar studies on ^3^He [6], ^4^He [7], D_2_ [8], and H_2_ [9]. The experimental cell consisted of three BeCu diaphragms welded at their circumferences and separated by 0.3 mm gaps. The lower gap, connected to a room-temperature He gas handling system via a capillary tube, had its pressure adjusted and measured directly. The upper gap was the T_2_ experimental chamber and was connected to the room-temperature T_2_ handling system via a low-temperature valve and a capillary tube. The sample pressure was determined from the deflection of the Upper diaphragm, measured by electric capacitance. The experimental volume was determined from the pressures in the two gaps, using the calibrations described in Ref. [8].

**Fig. 1:**
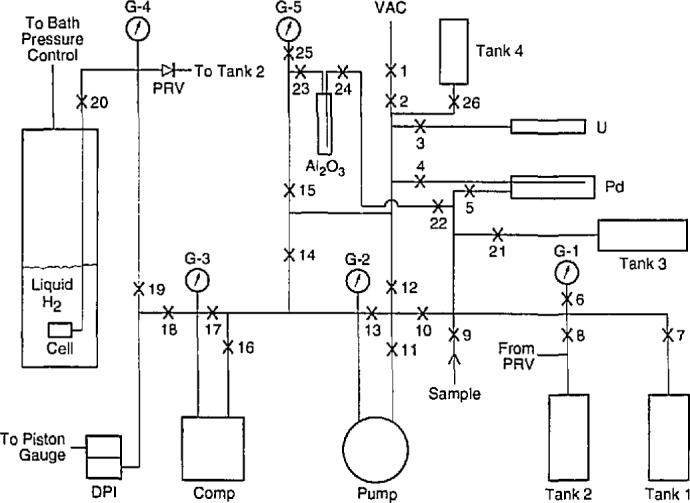
Schematic of tritium *PVT* system.

The T_2_ system is shown schematically in Fig. 1. Four stainless steel tanks, each of 1500 cm^3^ volume, were used to hold T_2_, either for storage or for transfer to various parts of the system. The T_2_ was pumped at low pressure with a rotary vane pump and compressed to 70 bar with a diaphragm compressor. The uranium bed (U), Pd diffuser (Pd), and AI_2_O_3_ adsorption tube (AI_2_O_3_) were used for T_2_ purification. T_2_ gas samples were collected in sample tubes and analyzed by mass spectrometer. Calibration of capacitance versus cell pressure was done with the cell valve (V20) open and the T_2_ separated from the oil piston gauge by a differential pressure indicator (DPI). To prevent excessive pressure in the cell upon loss of cooling when V20 was closed, a thermocouple on the cell signaled a motor to open V20, which allowed venting to a tank via a pressure relief valve (PRV). The plastic material in the cell valve tip and in the stem seals of the manipulative valves was the polyimide Vespel SP 211, which resisted the destructive action of T_2_ quite well.

## 3. Purification

A significant problem in T_2_ experiments is the growth of ^3^He from radioactive decay at the rate of 0.031% per day. It was anticipated that a ^3^He-T_2_ mixture would behave like a ^4^He-H_2_ mixture in solubility and effect on *PVT* measurements. The ^3^He growth during an experiment (at most 76 h long) was not expected to exceed solubility limits. Thus it was felt that the *PVT* measurements could be adequately corrected for ^3^He growth during an experiment but it was mandatory that the experiment start with ^3^He-free T_2_. Several methods of removing ^3^He were used. Exposure to U at 300 K binds T_2_ as UT_3_ and allows the unabsorbed ^3^He to be pumped away but good removal requires several cycles. A Pd tube diffuser retains all gases except the hydrogens. But these methods are slow and do not remove hydrogen and deuterium, which are initially present or appear in the gas when most materials are exposed to T_2_. Therefore the final process used was desorption from AI_2_O_3_, following basically the method of Depatie and Mills [10] for preparation of 99% o-H_2_ or p-D_2_. About 32 cm^3^ of 2 mm dia. pellets of AI_2_O_3_ was placed in a 21 cm long stainless steel tube (15.3 mm O.D. and 0.28 mm wall). At the center of this was a stainless steel tube (3.2 mm O.D. and 0.25 mm wall) for with-drawing the gas. Prior to use, the AI_2_O_3_ was evacuated at 140 °C for 2 h. The impure gas was added to the AI_2_O_3_ tube immersed in liquid H_2_ until saturation occurred at 87 mbar, after which it was passed through the tube at 87 mbar. The gas entered the top of the AI_2_O_3_ column and exited from the bottom until the exiting gas composition was the same as that of the entering gas, at which time flow was stopped. Then the liquid H_2_ bath was lowered slowly until the effluent gas was almost pure T_2_, after which the gas was collected separately while the adsorption tube warmed to room temperature. A pre- T_2_ test on D_2_ containing 0.61% HD produced 3500 cm^3^ STP D_2_ with 0.03% HD. For T_2_ initially containing 0.26% H_2_, 1.97% ^3^He, 7.34% HT, and 0.49% DT, Table 1 gives the composition of effluent gas samples taken at various points of withdrawal. Collection of the gas after *V*=1600 cm^3^ yielded 1600 cm^3^ T_2_ containing 0.18% H, 0.10% D, and < 0.01% 'He which was enough for a *PVT* run.

## 4. Ortho-Para Considerations

The equilibrium ortho-para composition in T_2_ for various temperatures was calculated by Jones [11] and Gaines, Tsugawa, and Souers [12] and measured by Frauenfelder, Heinrich, and Olin [13]. The Gaines et al. results (*T*≤ 22.5 K) agreed fairly well with the Jones results, which covered 0 K–175 K. The measurements [13] gave somewhat higher values of *c*, the *J* = odd concentration, which could result from a higher sample temperature than the thermometer reading because of the radioactive heating. The Jones calculation is used as the standard in this paper.

**Fig. 2:**
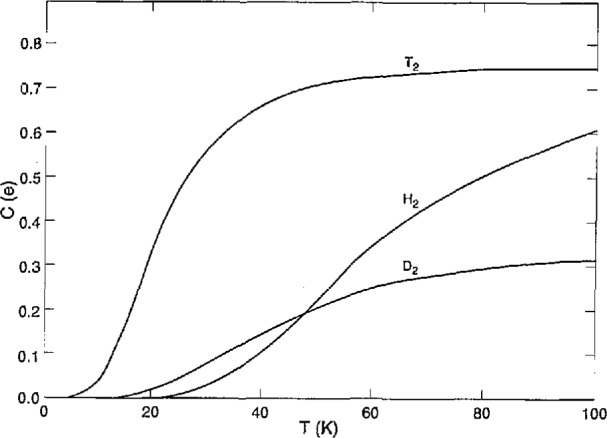
Concentration of *J* = odd states in the hydrogens at equilibrium vs temperature.

The equilibrium values *c* (e) versus temperature *T* for H_2_, D_2_, and T_2_ are shown to 100 K in Fig. 2. The normal (n) values (those at 7 = 300 K) are 0.75 for H_2_ and T_2_ and 0.33 for D_2_. While *c*(e) for H_2_ and D_2_ at 20 K is very small and insensitive to *T, c(e)* = 0.34 for T_2_ and increases rapidly with increasing *T.* Furthermore, the o-p conversion in T_2_ is much faster than in H_2_ under similar conditions. Therefore, it is important to determine *c* during the *PVT* measurements on T_2_. The variations of *c* with time *t* and vapor pressure were measured and partially reported earlier [14]. There, the values of *c* were derived from gas thermal conductivity measurements on samples from the condensed phase, thus the rapid back conversion, p→o, in the gas phase decreased reliability somewhat. The best fits of the data were: for the solid,

$$dc/dt=-kc^{2}$$ (1)

and for the liquid,

$$dc/dt=-k_{1}c^{2}+k_{2}c(1-c)$$ (2)

where *k* and *k*_1_ are empirical rate constants and *k*_2_=*k*_1_*c*(e)/(1−*c*(e)).

The results on o-p conversion are summarized in Table 2 in several useful forms: (a) *r*_0_, the conversion rate at zero time; (b) *t*_1/2_, the time to convert 1/2 way to equilibrium; and (c) *k,k*_1_, and *k*_2_, the rate constants. In solid T_2_, the observed *t*_1/2_ values of 2.0 h, 2.6 h, and 8.1 h at 4.0 K, 15.0 K, and 19.5 K, respectively, are moderately consistent with the NMR results of Gaines et al. [12] and Sater et al. [15] (although the latter found a minimum at 11.4 K) and with 1.5 h at 4 K of Frauenfelder et al. [13] using gas thermal conductivity analysis. However, Albers, Harteck, and Reeves [16] measured 0.28 h at 4 K with gas thermal conductivity. Our observed *t*_1/2_=8.3 h in liquid at 20.7 K also agrees with the Gaines et al. result. Thus, the conversion rates are about the same in liquid and solid near the triple point (20.6 K), which simplifies interpreting the *PVT* measurements on a liquid/solid mixture. The value of *k*_1_~8 × 10^−2^h^−1^ is eight times the *k*_1_ for H_2_ given by Woolley, Scott, and Brickwedde [17].

Vapor pressures of T_2_ at a certain *c* value, *P*(*c*), and of n-T_2_ *P*(n), were measured simultaneously in a special two-cell system. The differences, *ΔP=P(c)−P(n)*, are summarized in Table 3. Their behavior follows that of H_2_ at similar values of *P*(n), as in Woolley et al. [17]. For example, ex-trapolation of the *P*(n) = 840 mbar data to *c*=0 gives *ΔP =29* mbar for T_2_ and *ΔP* = 31 mbar for H_2_.

In the *PVT* measurements above vapor pressure, gas thermal conductivity could not be used to determine *c*. Instead, the variations of melting pressure and liquid molar volume with time were used to determine o-p conversion rates. In these measurements, it was assumed that the initial value of *c* was 0.75 because: (1) the purification process left the T_2_ sample at c ~0.75; and (2) the typical 2 h–5 h storage times at 300 K and 1.1 bar in a 1500 cm^3^ SS tank before condensation promoted conversion to n-T_2_.

## 5. Results

The *PVT* measurements typically began 2 h–3 h after condensation and continued for 50 h–76 h. Usually a single loading of the cell at a given *T* was used to measure compressibility and thermal expansion of liquid and solid, melting pressure, and volume change on melting. The liquid was compressed by a diaphragm until freezing began, which required 2 bar–4 bar overpressure. After the cell pressure stabilized, the compression was slowly continued past completion of freezing, which was indicated by a rapid rise in pressure.

### 5.1 Melting Pressures

The melting pressures *P_m_* discussed here were the first-freeze values, obtained by extrapolation to zero amount of solid. If the compression was delayed, the increase in *P_m_* with time was attributed to o-p conversion and ^3^He growth. The o-p change seemed to follow Eq. (2) where *c*=0.75 − *ΔP_m_*/*q, ΔP*_m_=*P*_m_(*c*=0.75), *q=ΔP_m_*/(0.75−*c*), and *k*_1_ and *q* are constant at constant *T.* Measurements of *P*_m_ for ^4^He-H_2_ mixtures made up in the gas phase showed the regular effects of a slightly soluble gas and agreed fairly well with results of Bereznyak and Sheinina [18]. The mixture *P*_m_ increased 3 bar- 4 bar per 1% of ^4^He over the *P*_m_ range of 0 bar–70 bar. Since ^3^He formation in T_2_ is 1.29 × 10^−3^% per hour, it was expected that ^3^He dissolved in condensed T_2_ would increase *P*_m_ by 3.7 mbar–5.0 mbar h^−1^, which would necessitate small corrections. If saturation were exceeded, the ^3^He would probably act as an ideal gas, i.e., *V* varies as *P*^−1^. Thus, the correction would be 60 mbar h^−1^ at the, lowest *P*_m_ (2.4 bar at 20.55 K), and 3.4 mbar h^−1^ at the highest *P*_m_ (70 bar). The ^3^He growth in 76 h (the longest time after purification) is 0.098% whereas the ^4^He-H_2_ measurements in this cell and in Ref. [18] gave 0.16% ^4^He as the solubility limit at 2.4 bar and 14 K. It follows that ^3^He would be expected to stay in solution. However, it apparently had left solution at 20.55 K when *P*_m_ and liquid compressibility *β*_1_ were measured. Here the measured "*P*_m_" was 2.4 bar, whereas linear extrapolation from higher *T* gave *P*_m_ = 0. If the excess pressure all came from ideal gas ^3^He the solubility would be 0.046% ^3^He. Sherman (R. H. Sherman, personal communication) measured 0.077%, which would result in 0.098−0.077 = 0.021% ^3^He as gas at 0.97 bar, which would yield 1.4 bar as the real *P*_m_. For this sample, the measured *β*_1_ was 10 times "normal," i.e., values for T_2_ aged 2 h–4 h. Furthermore, T_2_ with 52 h–70 h ^3^He growth at 20.60 K and 20.65 K showed *β*_1_ to be 4–7 times "normal." These high *β*_1_ values must have been the result of gas in the cell. The ^4^He-H_2_ mixtures containing up to 1% ^4^He, but below saturation, never gave *β*_1_ values greater than 10% above pure H_2_ values. This throws suspicion on the high *P*_m_ and *β*_1_ results for T_2_.

Taylor [19] summarized some experiments on condensed T_2_ in which ^3^He had grown beyond the normal solubility limit. In liquid and solid T_2_ there was a lack of vapor pressure buildup consistent with the ^3^He production rate. In another case, analysis of successive aliquots of gas removed from aged liquid T_2_ showed the last liquid was ^3^He-rich. Supplementary evidence for ^3^He not appearing as gas was provided by electrical conductivity and magnetic susceptibility measurements. The formation of free ^3^He was visually observed by Hoffer (J. K. Hoffer, personal communication), who condensed DT near the triple point in a cylindrical cell with sapphire windows at the ends [20]. After 8 d as a liquid, the DT showed no bubbles. (They could not be hidden in the fill tube, for it entered the bottom of the cell.) After a freeze and a melt, the sample showed a bubble at the top of the cell with a volume that was ~ 1% of the ideal gas volume for 8 d ^3^He growth. A second freeze and melt produced the same bubble, which persisted for 3 d. Then, within 12 h the bubble grew to 100% of the calculated volume for 12 d ^3^He growth, taking up 20% of the cell volume. During the next three days no change in the bubble was seen, even after a freeze and a melt. The behavior of ^3^He grown in condensed T_2_ seems to be unpredictable.

**Fig. 3:**
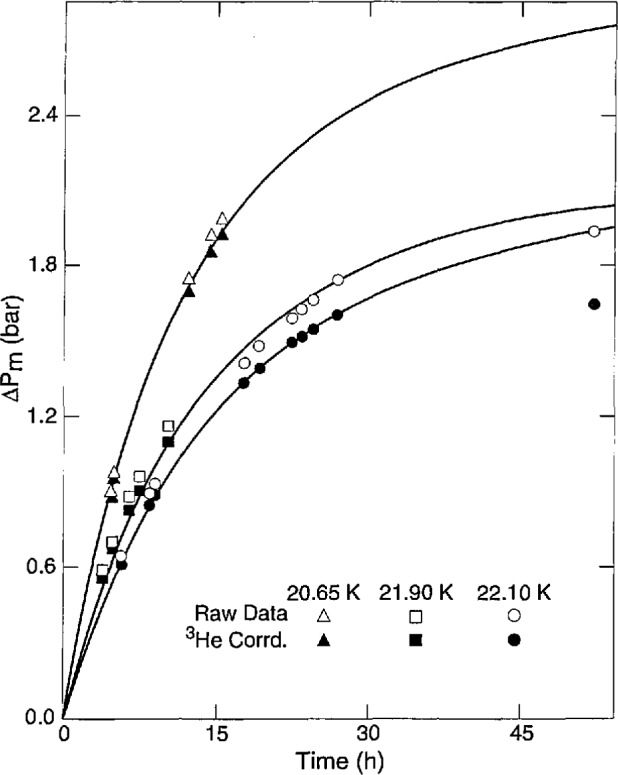
Tritium melting pressure vs time at several temperatures.

For this paper, the *P*_m_ measurements were corrected as if the ^3^He–T_2_ sample formed a solution like ^4^He-H_2_. Figure 3 shows *ΔP*_m_*=P*_m_(*t*)*−P*_m_(*0*) at 20.650 K, 21.900 K, and 22.100 K with and without ^3^He corrections. If the high rate at 20.65 K was caused by ^3^He growth, it seems that a greater correction is needed. In the fit to Eq. (2)*q* and the initial *ΔP*_m_ were varied to get the most consistent *k*_1_ for each run. The results, summarized in Table 4, show the similarities with the time variation of *c* in liquid at vapor pressure (Table 2). The average value *q* = 6.0 agrees with the H_2_ values 5.7–6.4 over 14 K–16 K from: *P*_m_ (p) by Youngblood [21]; *P*_m_(n) by Mills and Grilly [5]; and *P*_m_(p) and *P*_m_(n) in the present apparatus.

**Fig. 4:**
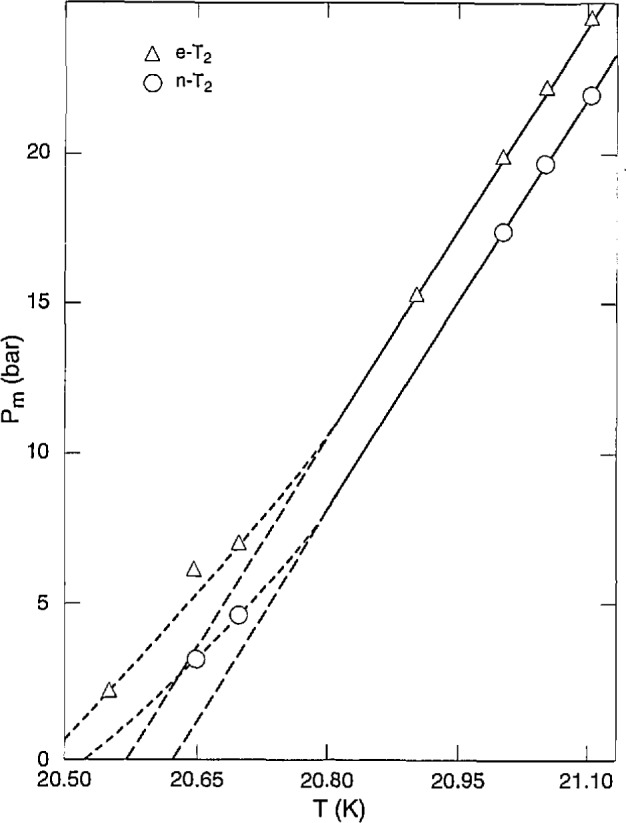
Tritium melting pressure vs temperature.

Regardless of the previous discussion, extrapolation *of P*_m_(*t<6h*) to *t* = 0 gave *P*_m_(n). For e- T_2_, values of *P*_m_(e) with ^3^He corrections were obtained from *q* values or *P*_m_(*t*~50 h). Corrections for 0.08%–0.42% H content (H_2_% + 1/2HT%) were made at the rate of −1.7 bar per 1% H. The results are summarized in Table 5, illustrated in Fig. 4 for *P*_m_ < 25 bar, and, over 20.83 K–22.10 K, fit the equations:

$$P_{m}(n)=0.22+45.92(T-20.627)\;\text{bar},$$ (3)

$$P_{m}(e)=0.22+45.27(T-20.568)\;\text{bar}.$$ (4)

The constant 0.22 is the triple point pressure for n-T_2_ determined from vapor pressure measurements [3], and it is assumed for e-T_2_ as well. The linear *P*_m_*–T* relation corresponds with the H_2_ and D_2_ curves. The greater values just above the triple point, as shown in Fig. 4, cannot be resolved at present. If Eqs. (3) and (4) held down to the triple point, the *T*_tp_ values would be 20.627 K for n-T_2_ and 20.568 K for e-T_2_. The 20.627 K value is close to *T*_tp_(n-T_2_) = 20.62 K, the junction of the liquid and solid vapor pressure equations [3]. Unfortunately, those equations ignored the highest point measured in the solid region, 2.116 bar at 20.547 K. If the liquid and solid curves went through that point, *T*_tp_(n-T_2_) would be 20.547 K, falling between the possible values from the melting curve, 20.53 K and 20.62 K. The *T*_tp_(e-T_2_) seems to be in the 20.48 K–20.57 K range. In Table 5, *P*–*P*_eq_ is the difference between experimental and equation values of *P*_m._

The sole previous *P*_m_ measurement in the present range was 56.68 bar at 21.826 K for n-T_2_ by Mills and Grilly [5], which is 1.48 bar higher than the present result. Of this deviation, 0.76 bar could be from the 0.9% HT impurity in the earlier measurement. Their equation gives values that are lower than the present by 2.5 bar. An equation devised by Goodwin [22] gives values lower than the present by 0.64 bar.

### 5.2 Volume Change with Time

**Fig. 5:**
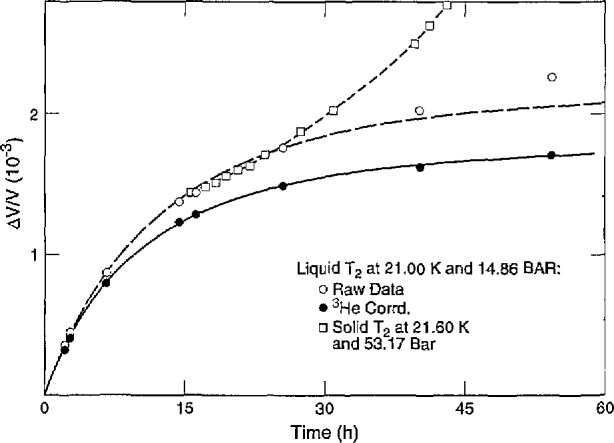
Relative volume change of T_2_ vs time.

The increase with time seen in liquid volume *V*_1_ at constant *T* and *P*(*~P*_m_) was also attributed to o-p conversion and ^3^He growth. The data fit Eq.(2) where *c=0.75−ΔV*_1_*/sV*_1_,* ΔV*_1_=*V*_1_(*c*)−*V*_1_(*c* =0.75), and *s = ΔV*_1_/(0.75−*c*)*V*_1_ with *k*_1_ = 7.53 × 10^−2^h^−1^ and *s* = 5.7 × 10^−3^ for the raw values of *ΔV*_1_/*V*_1_ at 21.00 K (*c*(e) = 0.368) and 14.86 bar. In Fig. 5, the raw data deviate from the dashed equation curve, indicating that o-p conversion almost stops after 30 h and thereafter *V*_1_ increases mostly from ^3^He growth. An empirical correction to *ΔV*_1_/*V*_1_, −1.0×10^−5^h^−1^ to yield coincidence between the corrected data and the solid equation curve results in *k*_1_ =8.98×10^−2^h^−1^ and *s*=4.6×10^−3^. The *k*_1_ values are similar to the results from *P*_m_ (Table 4), but the *s* values are smaller than the values for H_2_: 6.5×10^−3^ by Scott and Brickwedde [23] at vapor pressure; 6.7×10^−3^ by Wallace and Meyer [24] at *P*_m_. Measurements of *ΔV/V* vs *t* on solid T_2_ at 21.600 K and 53.17 bar were begun after the sample had been liquid for 6 h and solid for 9.5 h. They were added to the 21.000 K liquid value at *t* = 15.5 h. The results, shown in Fig. 5, follow the liquid curve for 9 h before rising sharply, probably because of breakup of the solid.

### 5.3 Liquid Thermal Expansion and Compressibility

**Fig. 6:**
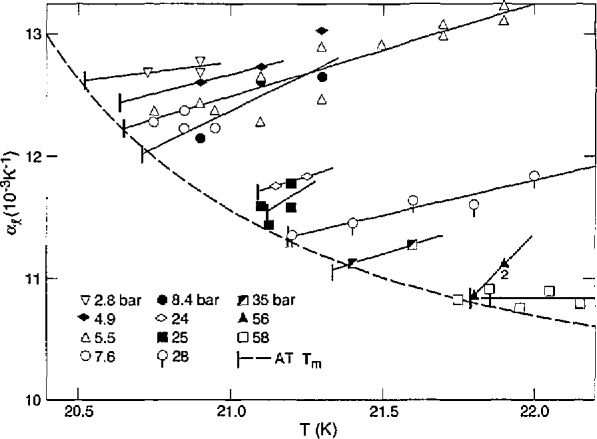
Thermal expansion of liquid e-T_2_ vs temperature at various pressures.

**Fig. 7:**
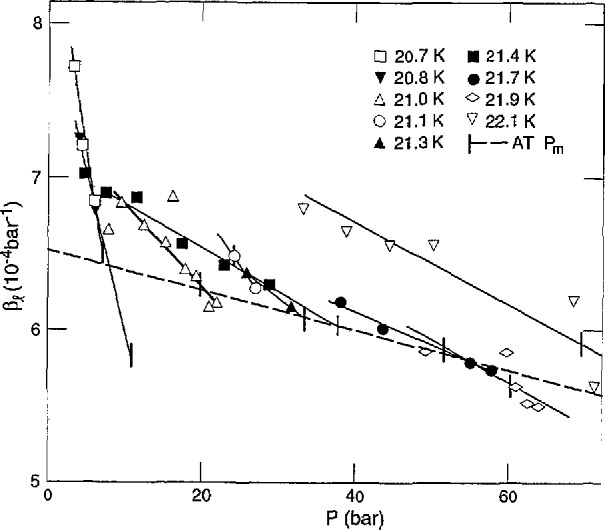
Compressibility of liquid e- T_2_ vs pressure at various temperatures.

The thermal expansion coefficient, *α = V^−1^*(*∂V/∂T)_P_*, and the compressibility coefficient, *β=−V*^−1^(*∂V/∂P)_T_*, of the liquid were measured directly. All *α* and 2/3 of the *β* measurements were made on essentially e-T_2_. The measurements at *c* = 0.6−0.7 fit in with the others. They would require a +1.5% correction, at most, for the volume change from o-p conversion during the 5 min measurement, and this is within the scatter of data. The differences in *α* and *β* for n-H_2_ and e-H_2_ were found to be within 2%. Therefore, it is assumed that the T_2_ data are independent of *c.* The *α* results are given in Fig. 6 as functions of *T* at various pressures. The dashed curve is through *T*_m_ of e-T_2_. The *β* results are shown in Fig. 7 as functions of *P* at various temperatures, and the dashed curve is through *P*_m_ of e-T_2_.

**Fig. 8:**
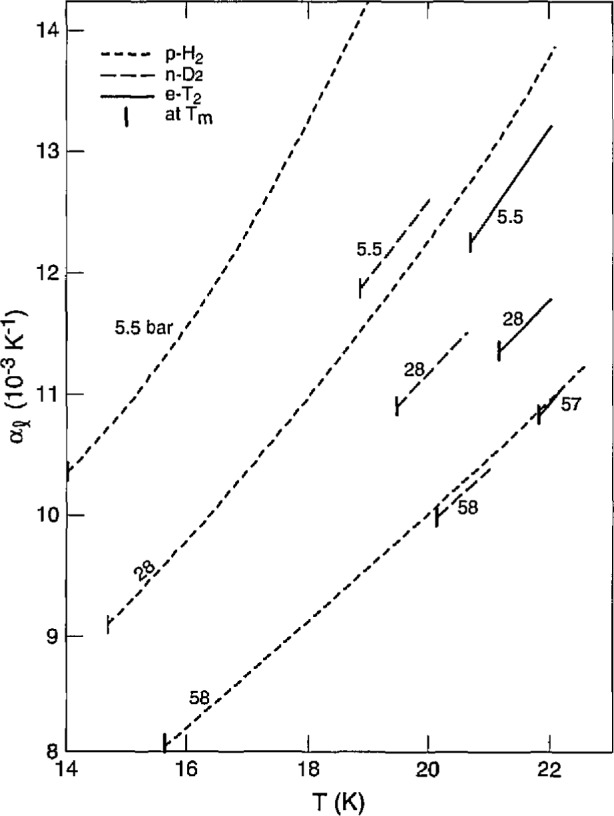
Liquid thermal expansion of the hydrogens vs temperature at several pressures.

**Fig. 9:**
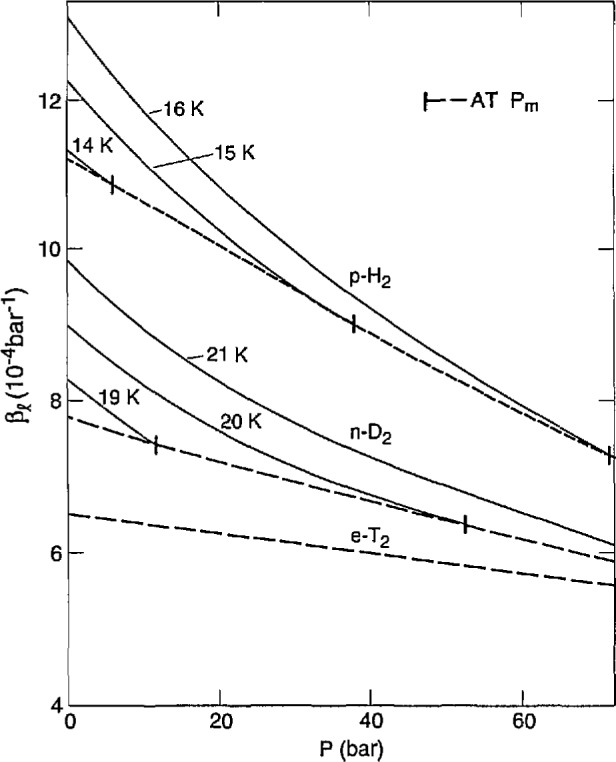
Liquid compressibility of the hydrogens vs pressure at several temperatures.

There are no other data on *α* or *β* for T_2_. Comparison of *α* for H_2_, D_2_ [8] and T_2_ is shown in Fig. 8. The three isotopes show similar slopes (*∂α/∂T)_P_* and their *α* values come together with pressure, becoming equal at 57 bar. Figure 9 shows *β* for the isotopes tending to merge at high pressures.

### 5.4 Molar Volumes

**Fig. 10:**
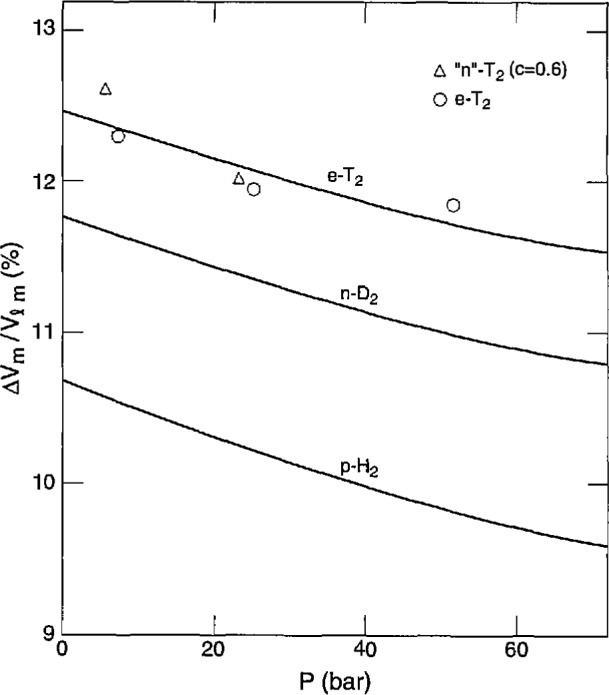
Relative volume change on melting of the hydrogens.

**Fig. 11:**
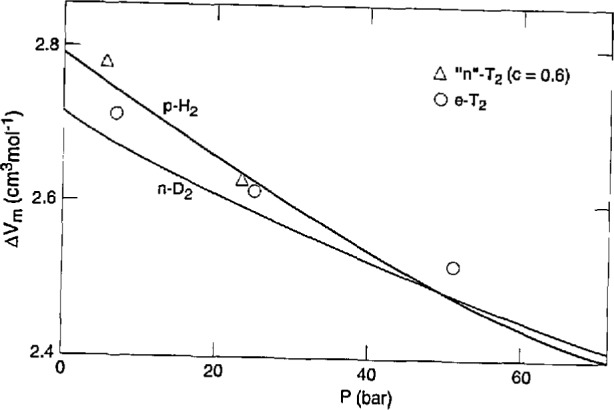
Volume change on melting of the hydrogens.

The molar volume of liquid T_2_ along the melting curve *V*_lm_ was calculated from the measurement at the triple point [4], 22.051 cm^3^mol^−1^ for n-T_2_, and the measured *α* and *β* values. This *V*_lm_ multiplied by the measured *ΔV*_m_*/V*_lm_ yielded *ΔV*_m_, the volume change on melting. Finally, the solid molar volume *V*_sm_ was determined from *V*_lm_ – Δ*V*_m_. Figures 10 and 11 give the results on *ΔV*_m_*/V*_lm_ and *V*_m_, respectively, for T_2_, H_2_, and D_2_ [8]. Essentially, the *ΔV*_m_*/V*_lm_ curves show a parallel displacement for the isotopes while the *V*_m_ curves are fairly close together. The results are given in Table 6. All the smoothed *PVT* values along the melting curve are summarized in Table 7 which should be self-consistent. Here, the *V*_lm_ and *V*_sm_ values are for n-T_2_, but the values for e-T_2_ are only slightly larger. Values of *V*_lm_(e-T_2_) – *V*_lm_(n-T_2_) were calculated from the o-p expansion and the *P*_m_ (n-T_2_)→*P*_m_(e-T_2_) contraction, using the *s* values in Table 4 and the *β* values in Table 7. The two effects largely cancel each other, leaving a net difference of only 0.002 cm^3^mol^−1^ for the most part, with high values of 0.009 at 20.535 K and 22.1 K. The result is carried over to *V*_sm_ since *ΔV*_m_/*V*_lm_ is assumed to be independent of *c*.

The possibility of comparison with other work is small. Hammel [25] predicted *ΔV*_m_=2.66 cm^3^mol^−1^ at the triple point, whereas here we get 2.734. Driessen et al. [26] calculated values of *V*_sm_ that are 0.05 cm^3^mol^−1^–0.07 cm^3^mol^−1^ lower than ours over 20.535 K–22.1 K range.

### 5.5 Solid Thermal Expansion and Compressibility

The measurements of *α* and *β* for the solid phase gave erratic and probably low values in general. This behavior can be expected from poor pliability of the solid in the measuring cell, which tends to be worse away from the melting curve [8]. The behavior occurred in all the isotopes, but T_2_ has other properties that could influence the measurements: ^3^He production, internal heating, and solid fracturing. Although the measurements were made on e-T_2_ the results can probably be used for any o-p composition.

**Fig. 12:**
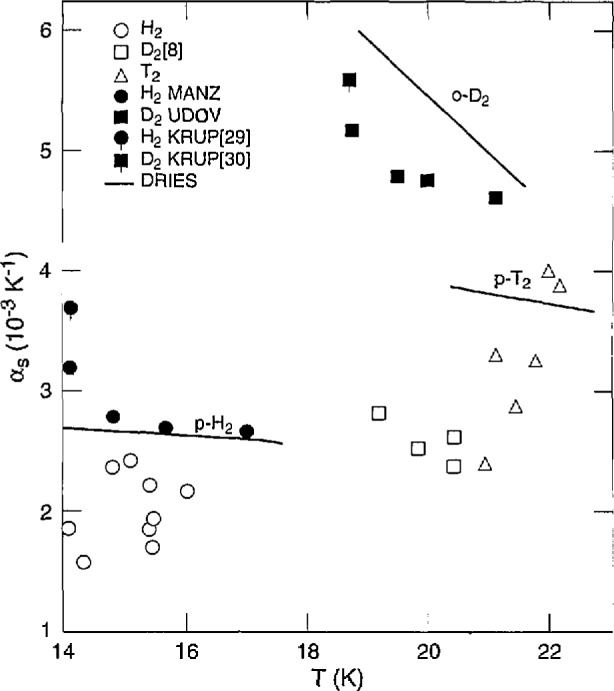
Solid thermal expansion of the hydrogens along the melting curve. MANZ is Manzhelii et al. [27]; UDOV is Udovidchenko et al. [28]; KRUP [29] is Krupskii et al. [29]; KRUP [30] is Krupskii et al. [30]; DRIES is Driessen et al. [26].

For each of H_2_, D_2_, and T_2_, *α* was measured at several pressures as a function of *T*, and each time it increased with *T.* However, *α* at constant *T* generally decreases with *P.* Thus the extrapolations of *α* to *T*_m_ can lead to roughly constant values, which occurs for H_2_ and D_2_ [8]. However ***α*** increases with *T*_m_ for T_2_. Figure 12 illustrates these behaviors, along with the overall increase in *α* from H_2_ to D_2_ to T_2_. The results of Driessen et al. [26] are also shown there. They measured the isochores of p-H_2_ and o-D_2_ up to 2 kbar, between the melting curve and 4.2 K, by use of a cell whose wall deflections were measured with strain gauges. Molar volumes were determined by correlation with data at the melting line and 4.2 K. Isochores were fit by integration of specific heat. The resulting equation of state was used to calculate *V*, *α*, and *β* up to 25 kbar. The derivation of an EOS for p-T_2_ was "guided by experimental results for H_2_ and D_2_." Their *α* results appear to be in rough agreement with ours for H_2_ and T_2_ but for D_2_ they are about twice as great. Densities were derived from dielectric constant measurements on p-H_2_ by Manzhelii et al. [27] and on n-D_2_ by Udovidchenko et al. [28]. Their *a* results (good to ± 10%), shown in Fig. 12, match the Driessen et al. results for H_2_ very well and for D_2_ within 15%. From x-ray studies of lattice parameters, Krupskii et al. [29,30] derived *a* for p-H_2_ that is 37% higher than the Driessen et al. result and *α* for o-D_2_ that is 8% lower.

**Fig. 13:**
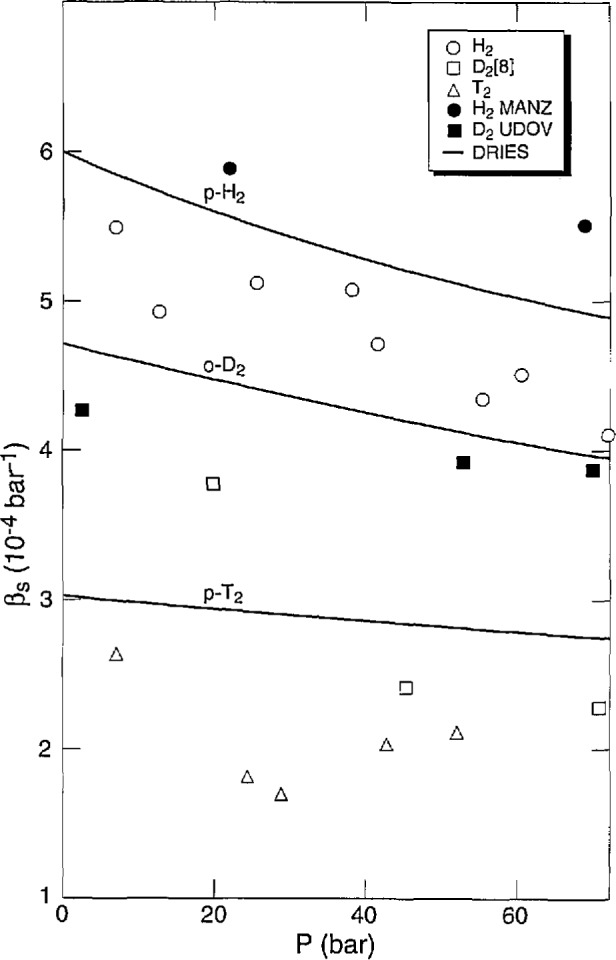
Solid compressibility of the hydrogens along the melting curve. MANZ is Manzhelii et al. [27,31]; UDOV is Udovidchenko et al. [28]; DRIES is Driessen et al. [26].

The measurements of *β* as a function of *P* at several temperatures show a decrease with *P.* Generally, *β* increases with *T*, therefore, the extrapolated values of *β* to *P*_m_ can be almost constant, as illustrated in Fig. 13. There is also a big decrease in *β* from H_2_ to D_2_ to T_2_. The values for H_2_, D_2_ [8], and T_2_ are about 0.90, 0.55, and 0.77, respectively, of the Driessen et al. [26] results. The measurements of Manzhelii et al. [27] and of Udovidchenko and Manzhelii [31] on *β* of p-H_2_ are 5%–10% greater than those of Driessen et al. [26] while the values of Udovidchenko et al. [28] for n-D_2_ are slightly lower. Other measurements on H_2_ and D_2_ were made at 4.2 K using various direct and indirect techniques. In general, the values are low. In some cases, values of P were not low enough to allow satisfactory extrapolations.

In spite of these discrepancies in *α* and *β* results, there is hope for more accurate values for T_2_. Overall, the Driessen et al. [26] results on H_2_ and D_2_ fit in fairly well with others. It follows that their T_2_ results should be credible. For example, the change in V_s_ along the melting curve between 20.5 K and 22.1 K is calculated from their *α* and *β* values to be 0.252 cm^3^mol^−1^, in reasonable agreement with *ΔV*_s_=0.287 in Table 7.

In solid H_2_ and D_2_, some anomalies in *α* and *β* were observed [8] but hardly deserve recognition as phase change effects. There is no point adding to the confusion in this subject [27, 28, 29, 30, 32, 33]. In T_2_, no anomaly was recognized, but observation was very limited.

### 5.6 Thermal Results

The enthalpy change on melting (heat of fusion) calculated from the Clapeyron equation *ΔH*_m_ = *TΔV*_m_d*P*_m_/d*T*, using the present *PVT* measurements on n-T_2_, is almost constant at 255 Jmol^−1^ in the range 20.9 K–22.1 K or 13 bar–70 bar. However, below 20.9 K the rapid decrease in d*P*_m_/d*T* lowers it to 144 Jmol^−1^ at *T*_tp_. On the other hand, Δ*H*_m_ for H_2_ and D_2_ varies linearly with *P*_m_ over 0 bar–70 bar from 117 Jmol^−1^ to 130 Jmol^−1^ for p-H_2_, according to Dwyer et al. [34], and from 197 to 210 for n-D_2_ [8], If we wish to focus more on the similarities of the isotopes, perhaps it would be better to compare the behavior of the entropy change *ΔS*_m_ *= ΔH*_m_/*T.* This decreases over 13 bar–70 bar by 2% for H_2_ and D_2_ and by 4% for T_2_.

## 6. Summary

The *PVT* relations in liquid and solid T_2_ were measured near the melting curve over 20.5 K–22.1 K and 0 bar–70 bar. They were compared with measurements on H_2_ and D_2_ and with calculations on T_2_. Comparison of the three isotopes leads to few surprises. The melting pressure variations with temperature and ortho-para composition are consistent. An exception is the strange behavior of Pm for T_2_ in the 0.3 K interval just above the triple point. The o-p conversion in condensed T_2_ is faster than in H_2_ but slow enough to allow observation of its effect on the *PVT* relations. The liquid and solid molar volumes of the three isotopes are consistent in magnitude and in their variations with o-p composition, pressure, and temperature. Still unresolved is the status of 'He produced in condensed T_2_.

## Figures and Tables

**Table 1:** Gas composition (%) as a function of effluent volume

*V*(cm^3^STP)	600	900	1200	1600	3200
H_2_	3.71	0.080	0.064	0.046	0.028
^3^He	0.90	0.010	<0.01	<0.01	<0.01
HT	75.84	5.66	0.36	0.38	0.20
DT	2.50	0.54	0.46	0.36	0.06
T_2_	17.05	93.62	99.12	99.21	99.72

**Table 2:** Ortho→para conversion in T_2_ at vapor pressure

Solid			

*T*(K)	4.0	15.0	19.5
*c*(e)	0	0.166	0.325
*k*(h^−1^)	0.675	0.321	0.065
*r*_0_(%h^−1^)	38	18	4
*t*_1/2_(h)	2.0	2.6	8.1

Liquid			

*T*(K)	20.7		24.4
*c*(e)	0.358		0.460
*k*_1_(10^−2^h^−1^)	8.46		6.32
*k*_2_(10^−2^h^−1^)	4.72		5.40
*r*_0_(%h^−1^)	3.9		2.5
*t*_1/2_(h)	8.3		8.8

**Table 3:** Vapor pressure of T_2_ at various ortho values

Phase	*P*(n) mbar	*T* K	*c*	*ΔP* mbar	*ΔP/Δc* mbar
liquid	840	24.4	0.66	6.0	67
liquid	840	24.4	0.54	12	57
liquid	840	24.4	0	(29)	(39)
liquid	228	20.7	0.48	4	15
solid	123	10.5	0.33	5.9	14
solid	116	19.4	0.46	4	14

**Table 4:** Ortho→para conversion in T_2_ from melting pressure change. Results in parentheses are from data uncorrected for ^3^He

*T*(K)	20.65	21.90	22.10
*P*_m0_(bar)	3.295	58.630	67.906
*c*(e)	0.358	0.393	0.400
*k*_1_(10^−2^h^−1^)	7.90(8.00)	6.58(7.00)	5.46(5.58)
*k*_2_(10^−2^h^−1^)	4.41(4.46)	4.26(4.53)	3.64(3.70)
*r*_0_(%h^−1^)	3.62(3.66)	2.90(3.09)	2.39(2.44)
*t*_1/2_(h)	8.86(8.74)	9.89(9.30)	11.76(11.54)
*q* (bar)	7.4(7.6)	6.1(6.2)	6.1(6.5)

**Table 5:** Melting pressure of T_2_

*T* K	*H*%^a^	*P(n)* bar	*P–P*_eq_ bar	*c*(e)	*t*^b^ h	*P*(e)^c^ bar	*P−P_eq_* bar	*q* bar
20.550	0.13			0.356	71	2.145^d^		
20.650	0.13	3.295^d^	+ 2.02	0.358		(6.196^d^)	+2.26	7.4
20.700	0.21	4.722^d^	+ 1.15	0.359	52	7.053^d^	+ 0.86	6.0
20.900	0.08			0.365	42	15.235	−0.01	6.4
21.000	0.08	17.419	+ 0.07	0.368		(19.864)	+0.09	6.4
21.000	0.21			0.368	68	19.772	0.00	6.2
21.050	0.22	19.632	−0.01	0.370		(22.140)	+ 0.10	6.6
21.100	0.42	21.968	+0.03	0.371	53	24.469	+ 0.16	6.6
21.390	0.15	35.198	−0.06	0.379		(37.239)	−0.19	5.5
21.600	0.35	44.660	−0.24	0.385				5.6
21.700	0.10			0.388	56	51.593	+ 0.12	5.8
21.750	0.25	51.751	−0.04	0.389	58	53.377	−0.35	4,5
21.900	0.10	58.630	−0.05	0.393				6.1
22.100	0.15	67.906	+ 0.05	0.400		(70.041)	+ 0.48	6.1
22.100	0.15			0.400	52	69.574	0.00	4.8

a Corrections to *P* were made at the rate of −1.7 bar for 1% H.

b *t* was the time in the condensed state when *P*(e) was measured.

c Values in parentheses are from Eq. (2) fitting.

d See text for the uncertainty involved.

**Table 6:** Volume change on melting of T_2_

*T* K	*P*_m_^a^ bar	Δ*V*_m_/*V*_lm_^a^ *%*	*V*_lm_ cm^3^mol^−1^	Δ*V*_m_ cm^3^mol^−1^	*V*_sm_ cm^3^mol^−1^
n-T_2_					

20.700	4.72	12.62	22.024	2.779	19.245
21.100	22.23	12.02	21.881	2.630	19.251

e-T_2_					

20.700	7.05	12.30	22.026	2.709	19.317
21.100	24.47	11.95	21.883	2.615	19.268
21.700	51.59	11.86	21.659	2.569	19.090

a Direct measurement.

**Table 7:** Properties of T_2_ along the melting curve

*T* K	*P*(n-T_2_) bar	*P*(e-T_2_) bar	*c*(e)	*α*_1_ 10^−3^ K^−1^	*β*_1_ 10^−4^ bar^−1^	*V*_1_(n-T_2_) cm^3^mol^−1^	*V*_1_(e-T_2_) cm^3^mol^−1^	*ΔV*_m_/*V*_1_ *%*	*ΔV*_m_ cm^3^mol^−1^	*V*_s_(n-T_2_)^b^ cm^3^mol^−1^
20.535^a^	0.22	1.80	0357	12.4	6.85	22.051	22.060	12.40	2.734	19.317
20.700	4.72	7.05	0.359	12.2	6.79	22.024	22.026	12.35	2.720	19.304
20.800	8.30	10.80	0.362	12.0	6.72	21.995	21.997	12.29	2.703	19.292
20.900	12.76	15.24	0.365	11.9	6.65	21.959	21.961	12.22	2.683	19.276
21.000	17.35	19.77	0.368	11.7	6.59	21.920	21.922	12.15	2.663	19.257
21.100	21.94	24.30	0.371	11.6	6.51	21.881	21.883	12.08	2.643	19.238
21.200	26.53	28.87	0.374	11.4	6.45	21.842	21.844	12.01	2.623	19.219
21.300	31.12	33.36	0.377	11.2	6.39	21.804	21.806	11.95	2.606	19.198
21.400	35.72	37.88	0.379	11.1	6.32	21.766	21.768	11.89	2.588	19.178
21.500	40.31	42.41	0.382	11.0	6.24	21.728	21.730	11.83	2.570	19.158
21.600	44.90	46.94	0.385	10.9	6.16	21.691	21.694	11.77	2.553	19.138
21.700	49.49	51.47	0.388	10.9	6.10	21.655	21.659	11.72	2.538	19.117
21.800	54.08	56.00	0.390	10.8	6.04	21.618	21.624	11.67	2.523	19.095
21.900	58.68	60.52	0.393	10.8	5.97	21.582	21.589	11.62	2.508	19.074
22.000	63.27	65.05	0.396	10.7	5.90	21.547	21.555	11.58	2.495	19.052
22.100	67.86	69.57	0.400	10.7	5.82	21.512	21.521	11.54	2.482	19.030

a See text on the triple point.

b *V*_s_(e-T_2_)−*V*_s_(n-T_2_)=*V*_1_(e-T_2_)−*V*_1_(n-T_2_).

## Footnotes

1 The bar (= 10^5^Pa) is used in this paper rather than the pascal in order to facilitate the comparison of the results of this work with the results of previous and similar work. It should be noted that the International Committee for Weights and Measures allows the use of the bar temporarily with the International System of Units.
